# A global patient outcomes registry: Cochlear paediatric implanted recipient observational study (Cochlear^™^ P-IROS)

**DOI:** 10.1186/1472-6815-14-10

**Published:** 2014-10-06

**Authors:** Georgina Sanderson, Thathya V Ariyaratne, Josephine Wyss, Valerie Looi

**Affiliations:** 1 Cochlear Limited, Asia Pacific Region, Macquarie University, 1 University Avenue, Sydney, NSW 2109, Australia; 2 Cochlear AG Europe, Middle East and Africa Headquarters, Peter Merian-Weg 4, 4052 Basel, Switzerland; 3 Sydney Cochlear Implant Centre, Macquarie University, Ground Floor, The Australian Hearing Hub, 16 University Avenue, Sydney, NSW 2109, Australia

**Keywords:** Hearing outcomes, Paediatrics, Cochlear implants, Bone conduction hearing systems, Prospective observational study, Patient registry, Quality of life, Multi-centre, Patient reported outcome, Multi-attribute utility index

## Abstract

**Background:**

Currently, there is a paucity of data concerning the long-term outcomes, educational placement and quality of life of children implanted with hearing devices from large and representative samples of the population. To address this concern, a large, prospective, multicentre, multinational patient-outcomes registry for paediatric recipients of implantable hearing devices was developed. The benefits of this registry, its approach and methodology are described.

**Methods/Design:**

The Cochlear^™^ Paediatric Implanted Recipient Observational Study (Cochlear P-IROS) is a prospective international patient-outcomes registry for children who are implanted in routine clinical practice with one or more hearing devices. The study aims to collect data on patient comorbidities, device use, auditory performance, quality of life and health-related utilities, across different types of implantable hearing devices from a range of manufacturers. Patients will be evaluated with a set of standardised and non-standardised questionnaires prior to initial device activation (baseline) and at six-monthly follow-up intervals up to 24 months and annually thereafter. The Cochlear P-IROS utilises a secure web interface to administer electronic case report forms to clinicians and families of implanted children. The web interface is currently available in five languages: English, Japanese, Korean, Mandarin and Russian. The interface also provides printable versions of the case report forms translated into 22 local languages for collection of data prior to entry online; additional languages may be added, as required. Participation in the Cochlear P-IROS registry is investigator-driven and voluntary. To date, the Cochlear P-IROS has recruited implant clinics across Australia, China, India, Indonesia, Turkey and Vietnam. The registry also aims to recruit multiple clinics in Cuba, Israel, Japan, Malaysia, Singapore, South Africa, South Korea and Russia.

**Discussion:**

The use of a registry such as the Cochlear P-IROS will generate valuable data to support research interests of academics and clinicians around the globe. The data generated will be relevant for a wide range of stakeholders including regulators, payers, providers, policy makers, patients and their families, each with a different perspective for the acceptance and adoption of implantable hearing devices for the treatment of hearing loss.

## Background

Hearing loss affects an estimated 5.3% of the world’s population that amounts to approximately 360 million people, of which 9% are children [1]. The global prevalence of hearing impairment, defined as an average hearing loss of 35 decibels hearing level (dBHL) or more in the better ear, is estimated at 1.4% in children aged 5–14 years [2]. The global prevalence of severe-to-profound hearing loss, defined as hearing loss of 61 dBHL or greater in the better ear [3], is estimated at 4.8% and 6.4% among children aged 0–1 years and 1–4 years, respectively (personal communication, G. Stevens, WHO). The prevalence of hearing loss varies across demographic regions and is associated with socioeconomic status, local risk factors such as exposure to infectious diseases, different cultural practices such as consanguinity and other causes also influenced by regional and geographic factors [2,4]. Prevalence of hearing impairment is greater in regions of low and middle income and is positively related to age and male sex [2].

The gold standard interventions available for treating patients with a permanent hearing impairment include cochlear implants and bone conduction implants. Cochlear implantation is the recommended treatment for children presenting with a permanent bilateral sensorineural hearing loss [5,6]. This impairment may range from moderate in the low frequencies sloping towards severe to profound in the high frequencies. Bone conduction implants may be prescribed for children with an ongoing permanent conductive hearing loss, mixed hearing loss or single-sided sensorineural hearing loss [7]. While these interventions remain the most widely used, in some jurisdictions where relevant regulatory requirements are satisfied, other implantable technologies such as middle ear implants or auditory brain stem implants may be recommended for children whose hearing loss is primarily due to malformations or injuries to the middle ear or the auditory nerve.

Large investments into research and development as well as over 30 years of clinical experience have established that cochlear implants and bone conduction implants are safe and clinically effective. This resulted in the expansion of the candidacy criteria to include very young children [8,9]. In the paediatric population, surgical risk from cochlear implantation is low and comparable to rates reported from general surgery for children [9-13]. A recent report inferred a major complication rate of 1.6% for children implanted bilaterally whose age ranged from six months to 18 years [14]. For bone conduction hearing implants in children, however, a number of factors, including the thickness of cortical bone, bone quality and skin thickness influence the surgical complication rate [15,16]. Compromised or soft bone with thickness <2.5 mm may present as contraindications for these devices in very young children. A recent meta-analysis reporting complications associated with bone conduction implants from various under-powered studies suggested rates in children ranging from 0.0% to 14.3%, 5.6% to 44.4% and 0.0% to 44.4% for osseointegration failure, implant infection and revision surgery , respectively [17].

Despite advancements in technology, there are currently few ‘best practice’ guidelines available to help inform professionals about implantable hearing devices [18]. Instead, there are a myriad of individual implant program protocols, each with variant indications developed over time, based on clinician expertise, published literature and services unique to each jurisdiction. The available data to support uniform evidence-based practice remains low and heterogeneous with relatively few studies being carried out in large and representative population samples with the purpose of collecting long term patient-related outcomes [17].

The National Institute of Health and Care Excellence (NICE) health technology appraisal into the clinical and cost-effectiveness of cochlear implants in adults and children, reviewed the body of evidence of cochlear implants with the consideration of expert opinion. The final appraisal document was published in February 2009 highlighting recommendations for clinical practice in the UK [19]. The NICE recommendations included the provision of simultaneous bilateral CI to children born deaf, adventitiously deaf or those newly diagnosed with severe-to-profound bilateral hearing impairment. The report also highlighted deficiencies in the available evidence, including difficulties in making comparisons due to the heterogeneity in measures and protocols, the absence of sufficiently long-term observation periods, the absence of benefits observed in daily circumstances and in the quality of life for the implanted child. To this end, the authors of the NICE report noted that more large scale studies are needed that prospectively follow-up patients for longer time periods, using standard measures of outcomes, and collecting full information of known covariates of post-implantation outcomes such as speech production, speech recognition and quality of life.

In response, a national audit was set up through collaboration of the cochlear implant programmes throughout the United Kingdom (UK). The UK national audit of paediatric bilateral cochlear implantation, which began in January 2010 and ended in December 2012, is one of the largest non-randomised prospective, observational studies exploring hearing outcomes in children with a permanent bilateral sensorineural hearing loss treated with cochlear implants [20]. The data collected included: listening ability, speech recognition, speech production, sound localisation, acquisition of vocabulary, parental perception and information relating to the implant surgery. Although the report from the audit showed encouraging results for bilaterally implanted children when considering hearing gains as well as acceptably low rates of adverse events, the study did not measure quality of life or other humanistic measures such as access to services, educational placement, literacy, numeracy or social inclusion, which are of interest to payers and policy makers.

With continued pressures on healthcare systems and budgets, there is now an increasing demand for evidence-based research that demonstrates an intervention is effective in the real-world environment across large and diverse populations. A common measure of utility such as ‘quality of life years gained’ is often requested by Health Technology Assessment (HTA) authorities, governments and payers so that a rational allocation of scarce resources may be achieved across the spectrum of healthcare interventions.

Many types of impairment have the potential to impact an individual’s everyday life, although the degree of impact may vary from one individual to another, independent of the severity of impairment [21]. Measuring not only the hearing-performance benefit of implantable hearing devices but also their impact on the individual’s everyday life (i.e., their quality of life) is, therefore, important. The WHO defines quality of life as an individual’s perception of their position in life in the context of the culture and value systems in which they live and in relation to their goals, expectations, standards and concerns [22]. Despite the availability of a number of instruments to measure an individual’s health-related quality of life, such as the EuroQoL and the SF-36, these remain limited in their sensitivity to hearing loss and their appropriateness for assessment of very young implanted children via parent proxy [23,24].

Several authors have recommended the use of a standardised, electronic patient registry for the collection of a homogeneous set of data for cochlear implant recipients [25,26]. Others have acknowledged that the implementation of an electronic registry across large and diverse recipient populations is both costly and complex [27]. Prospective, longitudinal patient outcome registries have also been proposed as an effective means to address the increasing demand for data on patient-related benefits of medical devices by the United States Food and Drug Administration (FDA) [28,29].

As a step towards generating a consistently collated and reported dataset on children implanted with hearing devices, the Cochlear Paediatric Implanted Recipient Observational Study (Cochlear P-IROS) was developed. This global, prospective, observational study provides for, and facilitates, a more unified approach for the assessment of patient outcomes cross-culturally, in a structured, sustainable and easily accessible format. The Cochlear P-IROS study design is based on the primary hypotheses of intra-subject improvements in auditory performance of children using implantable hearing devices across diverse language and cultural settings. A consistent set of questionnaire-based assessments and patient-related information will be collected via a web-based electronic data capture (EDC) platform.

The purpose of this paper is to introduce the design and methodology of the Cochlear P-IROS registry. The objectives of the study are threefold:

a) To evaluate the longitudinal improvements in auditory performance with implantable hearing devices in children using standardised questionnaires.

b) To provide statistically significant data to support patient management decisions at the clinical, regulatory, payer and policy level.

c) To compare the patient-related or humanistic benefits such as educational attainment, quality of life and patient satisfaction resulting from use of hearing implants in unilateral, bilateral and bimodal configurations.

## Methods/Design

### P-IROS synopsis

#### Study design

A prospective, longitudinal, observational study that will be implemented globally across multiple hearing implant centres on a voluntary basis. The study will be implemented through a secure, web-based, registry platform that enables collection of response data from clinicians and patient proxy via electronic case report forms (eCRFs) at consistent time intervals, thus enabling the comparison of repeated measures over time.

#### Population

The Cochlear P-IROS will enrol infants and children up to age 10 years, at the time of intervention. Children (referred to as ‘patients’), may receive any brand of regulatory approved implantable hearing device including any type of cochlear, bone conduction, electroacoustic or other implantable hearing device. If required, the Cochlear P-IROS platform also permits the capture of data from patients implanted at age 10 years or above, to address the growing trend for intervention in older children with an acquired or progressive hearing loss [30]. Additional questionnaires appropriate for this age bracket have been included for completion by these individuals' proxies. Patients above age 10 years may also be entered into the Cochlear IROS platform, a corresponding registry platform already implemented for adult patients.

The specific inclusion and exclusion criteria of the study are as follows:

Inclusion criteria:

1. Newly implanted prelingual and perilingual patients less than age 10 years, prior to the first time the hearing implant system is activated by the fitting of the sound processor.

2. Unilateral, bilateral or bimodal users of cochlear implants, electroacoustic devices, bone conduction implants, or other implantable hearing systems.

3. Parent/caregiver and/or patient are cognitively able to respond to self-administered/proxy assessment scales and willing to participate and sign the Patient Informed Consent form.

Exclusion criteria:

1. Patients with previous hearing implant experience. This includes all brands and types of cochlear, bone conduction, electroacoustic and auditory brainstem implant systems.

#### Study period

The Cochlear P-IROS registry will be available for clinics on a voluntary basis for monitoring the long-term progress of patients and sub-groups of patients using hearing devices. The recruitment of study sites is ongoing. Any implant clinic may join the Cochlear P-IROS to gather longitudinal data for newly implanted and enrolled patients at their discretion. For patients enrolled, baseline registration is scheduled post-implant, which may ideally be at any time after surgery and prior to the first time the hearing implant system is switched on by the fitting of the externally worn sound processor. Typically, activation occurs at two to four weeks post-operation. As illustrated in Table 1, suggested follow-up evaluations may be recorded at six months, 12 months, 18 months, 24 months, and annually thereafter for up to five years, ideally in parallel to routine clinical visits. Once a clinic voluntarily enrols a patient, it is assumed that adherence to the study protocol will occur, including a submission of their respective follow-up data for a minimum of two years post-surgery. Further evaluation of the patient, in the longer term, will remain optional. As the registry is entirely investigator-driven, the clinician is responsible for counselling and ensuring that the patient and their family are sufficiently motivated to participate in the study, long-term, while maintaining the decision for their participation as voluntary.

**Table 1:** Cochlear P-IROS evaluation schedule illustrating electronic case report forms used against the recommended evaluation time-point

**Electronic case report form**	**Evaluation time point (Months post-implant)**							
	**0**	**6**	**12**	**18**	**24**	**36**	**48**	**60**
Clinician baseline form	■	**-**	**-**	**-**	**-**	**-**	**-**	**-**
Clinician follow-up form	**-**	■	■	■	■	□	□	□
Implant recipient baseline form	■	**-**	**-**	**-**	**-**	**-**	**-**	**-**
Implant recipient follow-up form	**-**	■	■	■	■	□	□	□
Children using Hearing Implants Quality of Life (CuHI-QoL) Questionnaire	■	■	■	■	■	□	□	□
Health Utility Index Mark III (HUI3)**	■	■	■	■	■	□	□	□
Categories of Auditory Performance-II (CAP-II) Questionnaire	■	■	■	■	■	□	□	□
Speech Spatial Qualities (SSQ) Parents Version Questionnaire (SSQ-P)	□	□	□	-	□	□	□	□
Speech Spatial Qualities (SSQ) Standard Version**	□	□	□	-	□	□	□	□
Unaided hearing thresholds form	□	□	□	□	□	□	□	□
Aided hearing thresholds form	□	□	□	□	□	□	□	□
End of study form	**To be completed once when patient exits study**							

Notes:

**Will be available in Cochlear P-IROS if the patient is >10 years at time of implantation.

1. ■ represent required data capture (i.e., minimum dataset).

2. □ represent optional data capture.

3. – indicates that data capture is not relevant/required.

#### Ethical considerations

The Cochlear P-IROS will be conducted according to the guidelines established in the Declaration of Helsinki (Fortaleza, 2013) [31]. All study investigators, as well as the sponsor, are bound to follow obligations outlined in the Declaration of Helsinki and the ISO 14155:2011, the international standard regarding good clinical practice for clinical investigations of medical devices for human subjects [32]. The guidance includes the design, conduct, record and report, including protection of patient privacy for all clinical investigations performed in human subjects [31]. According to the standard, an *investigator* is defined as an individual member of the investigation team designated and supervised by the principal investigator (referred to as the ‘chief investigator’ in the Cochlear P-IROS) at an investigational site, to perform critical procedures or to make important decisions relating to the clinical study. The *sponsor* is defined as the organisation taking responsibility and liability for the initiation or implementation of the clinical study. Cochlear Limited is the sponsor for the Cochlear P-IROS (refer to Table 2).

**Table 2:** User roles by access levels to the Cochlear P-IROS and functionality

	**Investigator**				**Sponsor**		**Service provider**
**User (role)**	Chief investigator*	Unlimited investigator*	Limited investigator*	**Study nurse**	**Cochlear global project leader**	**Cochlear country project leader**	**Database administrator**
**Accepts terms of registry agreement**	√	-	-	-	-	-	-
**Obtain patient consent**	√	√	√	-	-	-	-
**Enter patient data**	√	√	√	√	-	-	-
**Edit patient data**	√	√	√	√	-	-	-
**View and download patient data**	√^1^	√^1^	√^2^	-	√ ^3^	√^4^	√^5^
**Transfer patient**	√	-	-	-	-	-	-
**Notified of new clinics and patients**	-	-	-	-	√	√	√
**Number of users permitted per study site**	1^**¥**^	≥1	≥1	≥1	NA	NA	NA

NA: Not applicable.

Notes:

√ indicates required functionality.

- indicates prohibited functionality.

* The term *clinician* used throughout the manuscript refers to the responsibilities of the: Chief Investigator, Unlimited Investigator and Limited Investigator. The Study Nurse user profile will be applicable for data entry purposes only and is, therefore, not typically a clinician.

^**¥**^ Each site requires one Chief Investigator, as a maximum. There is no upper limit to the number other investigators or study nurses that may be involved.

1. Can view/download data of all site’s patients.

2. Can view/download data of the investigator’s patients only.

3. Can view/download data of patients at the global level.

4. Can view/download data of patients at the national level.

5. Can only view patient data on request, for troubleshooting purposes, but cannot download patient data.

All sites wishing to participate in the Cochlear P-IROS must approach their responsible Ethic Committee (EC) or Investigational Research Board (IRB) for their formal opinion as to whether EC or IRB approval is required, or if a waver may be provided prior to commencement of any data collection. While the need for EC approval may be wavered, pending local or national jurisdictions, any centre planning to publish their data is recommended to obtain ethics committee approval, which is often requested as a pre-requisite for manuscript acceptance by scientific journal editors and reviewers.

Presently, the Cochlear P-IROS has gained approval from the ethics advisory board of Hear and Say (Brisbane, Australia), Peking University Third Hospital (Beijing, China) and the Istanbul University, Faculty of Medicine, (Istanbul, Turkey). In Turkey, one ethics approval from a local clinic was acceptable nationally; therefore ethics approval was waived at Marmara University Hospital (Istanbul, Turkey) and Osmangazi University Hospital (Eskisehir, Turkey). Ethics Committee approval was not required at the following study sites at the time of registration with the Cochlear P-IROS; First Affiliated University of Anhui Medical University (Hefei, China), Peking Union Medical College Hospital (Beijing, China), Saket City Hospital (New Delhi, India), Shruti ENT Hospital and Cochlear Implant Centre (Surat, India), Salemba Satu Medika (Jakarta, Indonesia) and Shandong Provincial Hospital (Jinan, China).

#### Patient informed consent

Patient informed consent is compulsory, and must be obtained in writing from a parent, guardian or the caregiver with legal responsibility for the implanted child, prior to the child’s enrolment in the registry. This is required after the patient is implanted, when the decision for device type and configuration has already been made, to ensure the decision to participate in the registry remains independent of the type and brand of the device implanted. A Cochlear P-IROS Patient Information Sheet will be provided to each participating family in their local language by the authorised investigators (refer to Table 2) who are responsible for obtaining patient consent and explaining the purpose of the study, why they have been chosen to take part in the study, potential advantages and disadvantages of participation, and what happens once the research study ends. Authorised investigators are also advised to review their local Patient Consent Forms to ensure that the information collected through the registry is in alignment with the terms and conditions for research in their clinic.

### Evaluation measures

The Cochlear P-IROS registry addresses the need for representative and uniform data concerning the clinical and humanistic benefits of patients using implantable hearing devices as medical evidence. Careful consideration was given to the selection of evaluation measures and their administration with respect to age at time of implantation of potentially recruited patients up to age 10 years. Factors considered included achieving a balance between subjective and objective measures, assessments of auditory performance that catered for the development as well as the range of listening and communication skills of the patients, suitable for administration via investigator and parental proxies, and suitable for translation and cultural adaptation [33].

The Cochlear P-IROS provides the guidelines and the evaluation tools for administration of both standardised and non-standardised sponsor-generated questionnaires for clinicians and parents/caregivers of patients using implantable hearing devices. Sponsor-generated questionnaires for clinicians and parents/caregivers were developed following a thorough review of the published literature for confounders of outcomes and in consultation with published researchers in the hearing implant field to address gaps in existing assessment of humanistic factors in children [34-39]. Through their repeated use in studies such as the Cochlear P-IROS, the collection of response data for these new questionnaires may support their standardisation over time. In addition to these subjective measures, pure tone averages of the implanted and non-implanted ears have been included. A summary of the full set of evaluation tools used in the study is provided in Table 3.

**Table 3:** A description of evaluation tools used in the Cochlear P-IROS for longitudinal assessment of speech perception, language development, auditory performance, educational placement, quality of life, device use and associated clinical and demographic covariates

**Construct**	**Evaluation tool**	**Reference**	**Categories of data collected**	**Scales used**
Clinical/surgical information, device-related data	Clinician Baseline Form	Sponsor generated forms	• implantable hearing device details	-
	Clinician Follow-up Form			
			• surgical approach used	
			• general medical and hearing history	
			• source of referral	
			• source of funding for implantation	
			• compliance to therapy/rehabilitation	
			• clinician satisfaction with progress	
Patient demographics, hearing history and habilitation	Implant Recipient Baseline Form	Sponsor generated forms	• hearing-aid use	
			• communication mode	
			• type of habilitation	
			• patient’s educational placement	
	Implant Recipient Follow-up Form		• family composition	
			• socio economic variables	
			• parent/carer satisfaction with child’s progress	
Auditory Performance	Categories of Auditory Performance-II (CAP-II) Questionnaire	[41]	Child’s functional use of audition in everyday situations	CAP–II score over 10-point scale (Range 0 to 9)
Self-reported hearing function	Speech Spatial Qualities– Parents’ Version (SSQ-P) Questionnaire	[38]	Auditory disability across a wide variety of domains, representing hearing in the everyday situations.	SSQ-P score over 3 domains
	Speech Spatial Qualities (SSQ) – optional for children 10 years or older	[36]	Auditory disability across a wide variety of domains, reflecting the reality of hearing in everyday situations.	SSQ score over 3 domains
Quality of life	Children using Hearing Implants Quality of Life Questionnaire (CuHI-QoL)	Sponsor generated form Additional file 1	The impact of hearing implants on the:	CuHI-QoL score over 3 domains
			• quality of life of very young children,	
			• their parents’ expectations and	
			• their immediate family.	
	Health Utility Index Mark III (HUI3) – optional for children 10 years or older	[48]	The HUI3 includes eight attributes of health status:	Health Utility Score (Range 0.00 to 1.00)
			• vision	
			• hearing	
			• speech	
			• ambulation	
			• dexterity	
			• emotion	
			• cognition	
			• pain and discomfort	
Unaided Hearing Threshold	Unaided Hearing Threshold Form	Sponsor generated form	• Otoacoutsic Emissions (OAE) and Auditory Brainstem Responses (ABR) may be recorded for very young children.	
			• Unaided air-conduction hearing-threshold measurements, pre- and post-implantation.	
			• Unaided bone-conduction hearing threshold measurements, pre- and post-implantation.	
Aided Hearing Threshold	Aided Hearing Threshold Form	Sponsor generated form	• Aided air-conduction thresholds, pre and post-surgery.	-
			• Aided bone-conduction thresholds, pre and post-surgery.	

#### Evaluation forms for the clinician

##### Clinician baseline form

This form serves for the registration of the patient at the baseline evaluation and focuses on the collection of patient profile specifics and their hearing history. It also collects information related to the implanted device/s and the external sound processors to be used, as well as any clinical details such as patient comorbidities, surgical approach, aetiology of the disease and hearing history. Questions in relation to number of weeks of gestation, physical balance, dizziness, commonly used mode of communication, physical and mental impairment, and other comorbidities and syndromes were included. Additionally, the questionnaire comprises questions on sources of referral and funds for implantation.

##### Clinician follow-up form

This form corresponds to the Clinician Baseline Form and is to be completed at each subsequent follow-up. The questions are designed to capture any changes that may have occurred since the previous evaluation interval, including details of any new implanted device/s and external sound processors. Additionally, it collects information regarding compliance to device use and therapy, the quality of family’s participation in the child’s habilitation and the clinician’s satisfaction with the patient’s progress [40].

##### The Categories of Auditory Performance – II (CAP-II)

The CAP is a widely-used, simple, language-independent, and culturally adaptable measure of auditory performance suitable for assessment in children of all ages, including patients less than age four years [37]. The majority of the literature, to date, has used the eight-point hierarchy rating scale of the CAP that assesses a child’s functioning in everyday situations. It covers a range of auditory performance abilities and also takes into consideration the different developmental rates of children. The CAP consists of a nonlinear, hierarchical scale with a score 0 equating to the lowest skill of, “no awareness of environmental sounds”, and a score 7 corresponding to the highest skill of, “use of telephone with a known listener”. Evidence shows a ceiling effect of the CAP as it does not address the more complicated listening skills now achievable with cochlear implants and other hearing devices, particularly when used in bilateral configurations. As such, an extension of the CAP was introduced in 2010, called the CAP-II [41].

The CAP-II, when compared to the CAP, adds two additional higher-level auditory skill categories to the original scale. As such, the CAP-II is a more sensitive measure of the range of auditory skills in hearing impaired children. Hence, the CAP-II was selected for use in the Cochlear P-IROS, to assess the patient-related auditory benefits arising from the use of implantable hearing devices in the unilateral and bilateral configuration compared with the pre-operative listening condition [14,20].

##### Unaided hearing thresholds form

This evaluation form collects unaided hearing thresholds measured through behavioural audiometry in the sound-field or via headphones for air-conduction and bone-conduction hearing thresholds performed routinely in the clinic. Thresholds for the speech frequency range 250 Hz 500 Hz, 1000 Hz, 2000 Hz, 4000 Hz and 8000 Hz are desired for measure and record for each ear. This form is used at the baseline visit following registration of the patient online, to capture the pre-implanted hearing thresholds for the patient. Subsequently post-operative hearing thresholds routinely measured for bone and air conduction in each ear may be recorded at follow-up intervals, using this form, as desired. For infants, where pure tone audiometry is not applicable, Otoacoutsic Emissions (OAE) and Auditory Brainstem Responses (ABR) may be recorded. Repeated hearing threshold measures may be compared over time to assess their stability following treatment.

##### Aided hearing thresholds form

This evaluation form collects aided hearing threshold levels for warble tones for each ear as routinely measured at the clinic through behavioural audiometry in the sound field, pre-operatively using a hearing aid and post-operatively using an implantable hearing device. Record of sound-field, aided hearing thresholds for warble tones in individual ears, where feasible, for the frequencies 250 Hz, 500 Hz, 1000 Hz, 2000 Hz, 4000 Hz and 8000 Hz, is desired at baseline and at follow-up intervals, six monthly intervals up to two years and annually up to five years post surgery. Repeated aided thresholds are compared over time and to unaided thresholds at the same time interval for determination of objective functional threshold gains.

##### End of study form

This form is completed for each registered patient upon his or her departure from the registry. This may be either at the completion of the prescribed set of evaluations (e.g., two, three, four, or five years post-implant) or prematurely at the request of the clinician or parent/caregiver or following an event requiring removal of the patient from further review. The clinician is asked to record the reason for premature termination of the review and subsequent data collection and date of exit from the study for each patient enrolled in the study.

#### Evaluation forms for the parent/caregiver

##### Implant recipient baseline/follow-up forms

These forms are self-administered by the parent/caregiver at baseline and follow-up intervals to collect complementary patient-specific data to that gathered in the Clinician Baseline/Follow-up forms. They are designed to gather basic hearing experience information and patient characteristics, including hearing aid use prior to and after the surgery, age at implantation, familial socio-economic factors, educational placement and communication mode, that may influence clinical and humanistic outcomes.

##### Children using Hearing Implants Quality of Life (CuHI-QoL)

This is a new Quality of Life instrument designed for this study, developed to assess quality of life of patients using implantable hearing devices via parent proxy (A copy of this questionnaire is available electronically) (Additional file 1). Its aim is to assess the impact of implantable hearing devices on the quality of life of very young patients, their parents’ expectations and the wellbeing of the family over time. A review of published literature on available health-related, quality of life instruments, hearing-health assessments and questionnaires used in the paediatric population and administered via parent proxy was undertaken during the development of this new measure. Specifically, an evaluation of the most commonly assessed domains and evidence of the corresponding sensitivity of the existing measures for implant treatment effects was reviewed. Published researchers of subjective scales used in the hearing-implant field were consulted to help determine the most relevant domains, content, and construct for this tool [42-48]. The resulting 25-item parent-reported questionnaire is divided into three sections; 1) Parental Expectations, 2) Impact on the Family, and 3) Quality of Life of the Child. The 25 questions cover the following domains; self–reliance, wellbeing and happiness, social functioning, general functioning, parental stress, and family cohesion. The questionnaire has been piloted to assess the feasibility and practicality of its use in multiple cultural (i.e., English and Asian) and socio-economic settings by parents and providers before being introduced as a tool for use in the study. The CuHI-QoL instrument is currently being validated by researchers at the Department of Audiology and Speech Therapy, University of Melbourne, Australia. The validation study includes parents of children with a hearing impairment who use either hearing aids or cochlear implants, and normal-hearing peers.

##### The Health Utilities Index - Mark III (HUI3)

The HUI3 is a generic health status and health-related quality of life questionnaire that was shown to be a sensitive measure to demonstrate impact of medical treatments over time across eight domains: vision, hearing, speech, ambulation, dexterity, emotion, dexterity, cognition and pain [48]. A number of studies demonstrate its effectiveness to determine the utility gained from implantable hearing devices [49-51]. The HUI3 version used in this study is suitable in children as young as age six years [48]. A conservative approach was taken for the Cochlear P-IROS, making the questionnaire available for patients greater than or equal to age 10 years at the time of enrolment. Validated translations have been licensed and made available specifically for use in this study.

##### The Speech, Spatial and Qualities of Hearing Scale (SSQ)

The SSQ scale is a standardised questionnaire that was shown to be a sensitive measure to demonstrate benefits of bilateral versus unilateral hearing ability in a variety of daily situations and a range of hearing abilities, across three domains: speech, spatial and quality in adolescents and adults [36]. Compared with the CAP-II, it provides more finely detailed information about a patients’ auditory performance in everyday situations, allowing for detailed analysis. This version of the SSQ is available for completion by enrolled patients greater than or equal to age 10 years at the time of implantation.

##### The SSQ-Parents’ version (SSQ-P)

The SSQ-P is available for response via parent proxy for enrolled patients aged four to 10 years at the time of implantation. Based on the original SSQ above, covering the same three domains, it was modified and shortened for administration as a self-assessment tool for parental proxy following observation of their child’s behaviour in various listening environments in their daily lives, as considered applicable [38]. The SSQ-P is not recommended for parental proxy of children less than age four years, as the developmental changes observed in these patients may confound the judgment and, subsequently, the measurement of intervention effect over time. The reasons include difficulties associated with accurately rating the performance of younger patients in questions such as, “following a group conversation” [38]. In the design of this questionnaire for use in non-English speaking countries, a validated translation process was employed (e.g., from English to Hindi and Hindi to English). Necessary cultural adaptations were also made as required (e.g., use of the term ‘motor scooter’ instead of a ‘lawn mower’ in India) through personal communications with William Noble (University of New England, NSW, Australia), the co-developer of the original SSQ.

### Study hypotheses

#### Primary hypothesis

a. Post-implant performance for all patients on the Categories of Auditory Performance-II (CAP-II) are superior to pre-implant performance (baseline) and show incremental improvement at each subsequent post-implant assessment point (six months, 12 months, 18 months, 24 months, and annually for up to five years) during the study.

#### Secondary hypotheses

a. Post-implant hearing ability for patients > 4 years of age as assessed via the standardised Speech Spatial and Qualities scale Parents’ version (SSQ-P) are superior to their pre-implant hearing ability (baseline) and show incremental improvement at each subsequent post-implant assessment point (six months, 12 months, 18 months, 24 months, and annually thereafter for up to five years) during the study.

b. Post-implant auditory performance for patients using binaural hearing/stimulation is superior to that of patients using unilateral hearing/stimulation at each post-implant evaluation point (six months, 12 months, 18 months, 24 months, and annually thereafter for up to five years) during the study as measured by assessments of the:

i. Categories of Auditory Performance – II (CAP –II) scale for all patients

ii. Speech Spatial and Hearing Qualities Parents’ version (SSQ-P) scale

iii. Post-implant, aided hearing-threshold levels

#### Tertiary hypotheses

a. Post-implant assessment of quality of life for the patient and family via the CuHI-QoL questionnaire as assessed by the parent or caregiver are superior to quality of life assessed at baseline (pre-implant) and show incremental improvement at each subsequent post implant evaluation time point (six months, 12 months, 18 months, 24 months, and annually thereafter for up to five years) during the study.

b. Patients who begin mainstream school during the study enter at an age-appropriate time.

c. The proportion of patients who are participating in mainstream school with no additional support is higher than the proportion of patients in other categories of school placement.

### Study implementation

#### Data management

The Cochlear P-IROS registry is managed by an experienced, third-party database service provider based in Gembloux, Belgium. The data is stored centrally in an externally hosted, electronic data capture (EDC) platform that is compliant with the standards of the International Conference on Harmonisation – Good Clinical Practice (ICH-GCP) and the US FDA-21-Code of Federal Regulations-Part 11 (FDA-21-CFR-part-11) [52-54]. The Cochlear P-IROS registry protocol adheres to published guidelines for the design and implementation of patient-outcomes registries [33,55]. The Cochlear P-IROS electronic, web-based platform is currently available in five languages; English, Mandarin, Korean, Japanese and Russian. The minimum hardware and software requirements for implementation of the registry include having an internet connection allowing normal and secured communications as well as Adobe Reader Version 8.0 or higher installed (Note: Safari 5.1 or later requires Adobe Reader 10.1.4 or higher.)

#### User access

Access to the information collected by the Cochlear P-IROS registry is limited by functionality to a confined number of users at the study site and Cochlear Limited, as defined in Table 2. Each site may have up to four types of investigators including one chief investigator and one or more of an unlimited investigator, limited investigator and a study nurse. The *unlimited* versus *limited* nature of an investigator’s responsibility is related to their capacity to view, edit and download all of the site’s patient details versus only the details of the patients they personally enrolled into the system. Data may be viewed and downloaded (but not edited) by the global and country level project leaders of the Cochlear P-IROS. The database administrators from the third-party EDC provider of the Cochlear IROS platform may also gain access to view data and troubleshoot, should problems arise. To gain user access, a user must complete the IROS User Request Registration Form. User access registration forms are available upon request through contacting the global project leaders of the study (GS, JW and TVA) at CochlearIROS@cochlear.com.

#### Data privacy and intellectual property

All clinics will have ownership rights for their site’s data and the flexibility to operate under their own local processes or regulations around data collection, privacy and maintenance of patient records. In Australia, all participating clinics must obtain ethical approval for the Cochlear P-IROS from a legislated Human Research Ethics Committee (HREC), who will assess the study’s protocol and documents for breach of privacy standards. Under the *Australian Privacy Act*, the data collected and stored by the Cochlear P-IROS registry is not classed as, “personal information”, as a patient’s identity is preserved within the database, i.e., no identifiable personal data is collected in any of the evaluation forms administered as part of the study. The Cochlear P-IROS registry is also listed on the Australia New Zealand Clinical Trials Registry, a public database for clinical research in humans that is globally recognised [56].

#### Electronic case report forms

The logical framework of the Cochlear P-IROS registry is set-up as a series of evaluation tools in the format of eCRFs that must be completed through data entry via the electronic platform according to the study’s evaluation schedule. The evaluation schedule highlighting the required and optional assessments is illustrated in Table 1. Paper copies of the eCRFs may also be downloaded and printed from the study platform at any time, ultimately for completion, and storage, ideally in the patient’s clinic file, until data entry online can be undertaken for electronic data capture.

To date, the case-report forms for patients have been translated into 22 languages spoken in China, Japan, Korea, India, Middle East, Taiwan, Turkey, South Africa, South East Asia, Europe and South America and Russia.

#### Data entry

The Cochlear P-IROS registry may complement management of patients at clinics including their routine clinical follow up. A clinician or another approved person at the clinic could enter the data into the Cochlear P-IROS platform. Investigators participating in the registry will have access to real-time patient data updates as well as automated summary reports through the use of their confidential, system-allocated password.

For investigators, automated email reminders notifying follow-up times for individual patients will be sent two months before the evaluation is due. If participating families wish to opt-out of the study, completion of an End of Study form will be required.

For parents/caregivers who have elected and agreed on the patient informed consent form to respond directly on-line, an automated email reminder will be sent two months prior to the next follow-up evaluation for their child, and on the day of the scheduled follow-up. If a parent/caregiver do not respond and complete the recommended evaluations for a particular time-point, the clinician will be subsequently notified by email. A notification will be sent to the clinician, once the parent has completed their data entry for that time-point.

#### Data outputs

The Cochlear P-IROS web interface facilitates centre-specific, automated summary reports on the number of patients recruited to-date, their age at implantation, number and types of implants and outcomes (i.e., mean CAP-II, mean SSQ-P and mean CuHI-QoL). Country and global summaries across these measures are also provided to support national and international benchmarking of performance. Routine newsletters and reports on the study progress and other updates are to be developed by the global administrators and sent to the participating centres.

#### Data monitoring

There will be no routine onsite monitoring undertaken of the data entered. Participating clinics are not asked to store paper documents as source documents for data entered online. Hence, cross checks between data entered and source data is not possible. Online random spot-checks will be conducted on five percent of the data entered quarterly at each participating Cochlear P-IROS clinic by the global administration team.

The platform has been designed with inherent checks-of-response fields entered on eCRFs and automatically pending response types. The vast majority of responses on all forms are multiple choice, check box, radio buttons or pull-down response options to facilitate entry and reduce entry error. Minimal free-text response fields are included. The electronic registry platform operating the Cochlear P-IROS contains an inherent audit trail to trace all amendments made with each form, the investigator making the change and when changes are made. To avoid unnecessary changes, eCRFs are automatically locked upon access and data entry into the corresponding form at a subsequent assessment interval. Manual unlocking of these forms can be requested by the clinician to the Cochlear P-IROS global administrator.

#### Statistical analysis

A proposal for the statistical methods and data analysis plan for the assessment of Cochlear P-IROS hypotheses created by a consultant statistician is available as a separate report upon investigator’s request. Prospective, longitudinal studies, such as the Cochlear P-IROS, are especially powerful due to the repeated measures on participants. As such, relatively low numbers of observations may be adequate for most tests. A sample size of n = 50 was considered sufficiently sensitive to measure implant treatment effects assessed by the CAP-II, based on published literature. Current publications on paediatric cochlear implants lack sufficient detail on the SSQ-P or SSQ to generate an accurate power calculation for the sample sizes needed to find significant differences or changes; whilst CuHI-Qol is a completely novel outcome measure and, therefore, a sample size calculation is not currently possible.

It is also not possible to determine the actual number of patients that will be registered with the Cochlear P-IROS over time, as participation in the Cochlear P-IROS is voluntary for the clinic and the parent/caregiver/patient. Additionally, the parent/caregiver/patient may also cease to participate in the registry at any time. Nevertheless, the design of the database does not pose any constraint on the number of patients that may be enrolled over time, as it was developed with the aim to sustain data collection in the long-term. Large scale enrolment of patients in the range from 100 to 1000 or more on an annual basis is expected.

## Discussion

The Cochlear P-IROS provides a unique opportunity for improving the evidence base relating to outcomes achieved by patients with a permanent hearing impairment using implantable hearing devices. It removes barriers to participation from non-English speaking clinicians and patients by offering an easy-to-access, secure web platform with multiple language options and automated reports to support benchmarking activities. The registry is designed to collect clinical, demographic and patient-related outcomes data from newly implanted patients who are presented for routine intervention.

The Cochlear P-IROS web interface, as well as patient forms, have been translated into multiple languages in order to enable cross cultural data collection and subsequently cross cultural application of the interpreted outcomes. Thereby, the study reduces the potential risk of selection bias that may arise from exclusion of patients with a non-English speaking background. The Cochlear P-IROS design also minimises potential bias from recall errors, with a recall memory period of four weeks requested at each evaluation interval [57]. The authors, however, acknowledge that there may be a risk of emotional bias for the baseline evaluation time point. This is because the evaluation takes place part way through the two-part fitting of an implantable hearing device; that is, after the surgical placement of the internal implant but before activation of sound, with the fitting of the external sound processor.

The registry addresses industry-wide concerns around a lack of language-independent, standardised outcome measures for paediatric recipients of implantable hearing devices. To date, the wide variety of language-based, auditory-related outcome measures utilised in this patient population hinders effective meta-analysis [19]. Through the development of a new quality of life instrument, the CuHI-QoL, and the use of a range of standardised outcome measures (SSQ-P and CAP-II), Cochlear P-IROS aims to provide a homogeneous set of data for the comparison of benefit of implantable hearing devices for considerably larger numbers of patients that are more representative of the treated population.

The advantages of using questionnaires for subjective assessment is that it allows for a view to the benefits obtained post-implantation through the patient’s eyes or in the eyes of their parents, specifically demonstrating hearing benefits and additional hearing-related benefits. A further advantage, not specifically addressed in this paper is the ability to use validated translations of such questionnaires for collection and collation of qualitative patient-reported benefits across different languages, both pre and post-implant, longitudinally [19,9,39]. Comparison of auditory benefit data assessed and reported using local speech audiometry measures is both challenging, in view of the differences in materials and methods, and not recommended where equivalency in materials and measurements is unknown. The Cochlear P-IROS approach could foster not only consistency of data collected but also encourage the reporting of consistent evaluation methods. This makes it one step closer to the possible availability of published meta-analyses of collective standardised outcomes from patients using implantable hearing devices, which could potentially be used by regulators, health technology assessors and health service provision decision makers.

The Cochlear P-IROS may preclude the need for large local capital investment to design and implement a multi-centre web-based registry. The study provides the grounds for various hearing implant clinics to develop and own their own set of patient profiles, outcomes data and accordingly pursue their own research interests which may lead to scientific publications. Collaborative, multi-centre publications are especially encouraged to combine data collection efforts from different investigators, which may strengthen the potential power of the data reported and the interpretation of the conclusions drawn. The Cochlear P-IROS registry, therefore, provides a cost-effective method for interested parties to establish and invest in the outcomes of patients using implantable hearing devices in the long term, in a clinically feasible, broadly consistent and practically sustainable manner.

## Abbreviations

AHRQ: Agency for healthcare research and quality; CAP-II: Categories of auditory performance – ii; CuHI-QoL: Children using hearing implants quality of life; eCRF: Electronic case report form; EDC: Electronic data capture; ENT: Ear nose and throat; FDA: Food and drug administration; HUI 3: Health utilities index mark III; IRB: Institutional review board; IROS: Implanted recipient observational study (for Adults); ICH-GCP: The international conference on harmonisation – good clinical practice; ISO: International organisation for standardisation; P-IROS: Paediatric implanted recipient observational study; SSQ: Speech, spatial, quality.

## Competing interests

The three authors GS, TVA and JW are current employees of the company Cochlear Limited. VL is an independent researcher and declares that she has no competing interests. The Cochlear P-IROS Registry is commercially funded by Cochlear Limited. Individual clinic participation, implementation and publication of outcomes are entirely voluntary. No sponsorship is offered for associated activities in the participating clinics.

## Authors’ contributions

GS, TVA and JW are the Global Administrators of P-IROS and are involved with project management including acquisition and interpretation of data on behalf of Cochlear Limited. GS, JW and VL were involved in the design of the registry concept and associated database development. TVA drafted the manuscript. GS, JW and VL edited the manuscript. All authors read and approved the final manuscript.

## Authors’ information

GS is the Director of Policy and Market Access at Cochlear Limited and holds the following qualifications: BSc (Hons), MBA, Grad Cert in Health Economics. TVA is a Health Outcomes Associate at Cochlear Limited and holds the following qualifications: BBMedSci (Hons)/ BEcon. JW is a Senior Clinical Studies Specialist at Cochlear Limited and holds a Dip Audiology and BSc. VL is the Senior Research Manager for the Sydney Cochlear Implant Centre, Sydney Australia and holds the following qualifications: PhD, M Clin Audiol, P/Grad Dip MusTh, BA-Mus, M Aud SA(CCP), RMT.

## Pre-publication history

The pre-publication history for this paper can be accessed here:

http://www.biomedcentral.com/1472-6815/14/10/prepub

## Supplementary Material

Additional file 1 **Children using Hearing Implants Quality of Life (CuHI-QoL) questionnaire.** This is a new quality of life instrument developed to assess quality of life of patients using implantable hearing devices via parent proxy. Its aim is to assess the impact of hearing implant devices on the quality of life of very young patients, their parents’ expectations and the wellbeing of the family, over time. Click here for file
