# Supplementary material for: A global patient outcomes registry: Cochlear paediatric implanted recipient observational study (Cochlear™ P-IROS)
Source: BMC Ear Nose Throat Disord. 2014 Oct 6;14:10. doi: 10.1186/1472-6815-14-10 (PMC4196206; doi:10.1186/1472-6815-14-10)
Supplement: Additional file 1 — Children using Hearing Implants Quality of Life (CuHI-QoL) questionnaire. This is a new quality of life instrument developed to assess quality of life of patients using implantable hearing devices via parent proxy. Its aim is to assess the impact of hearing implant devices on the quality of life of very young patients, their parents’ expectations and the wellbeing of the family, over time. [file 1472-6815-14-10-S1.pdf]

Children Using Hearing Implants Quality of Life Questionnaire

Subject ID

To be completed by researcher

Date completed ☐ / ☐ / ☐ **(dd/mm/yyyy)**

My child is a ☐ **boy** ☐ **girl**

My child's current age ☐ **years** ☐ **months**

Time since implant surgery ☐ **(months)**

I am the ☐ **mother** ☐ **father** ☐ **other**

This questionnaire aims to assess the effect of the hearing device(s) for your child's hearing loss; thinking about their hearing ability, their quality of life, the impact on your family and your expectations for your child over time. The questionnaire can be completed by you as the parent/ care giver, reporting on your own observations, and feelings about your child's development and everyday life.

This questionnaire has 25 questions, divided into three sections. It will be completed for the first time before your child starts using their hearing implant(s) to record your impressions about life without the device. The same questions will be repeated at regular intervals after your child starts using their device(s) so that you may report on how life has changed over time for you, your family and your child.

Please read each question carefully. For each question, please select one answer that best describes your experience with your child over the last 1-2 months. It would be greatly appreciated if you would respond to each question. Where a situation does not apply to you please select the 'not applicable' (N/A) option.

The questionnaire will take between 5 - 10 minutes to complete all 25 questions.

All information you provide is confidential. There are no right or wrong answers; what we want is your opinion.

We would like to acknowledge that this questionnaire has been developed with the advice and input from:

- Dr Sue Archbold, Chief Executive, The Ear Foundation, Nottingham, United Kingdom

- Dr Dimity Dornan, Managing Director, The Hear and Say Centre, Queensland, Australia

**SECTION 1**<br>**Expectations for Your Child**	Strongly Agree	Agree	Unsure	Disagree	Strongly Disagree	N/A
1. I believe my child will easily make friends with other children.	☐	☐	☐	☐	☐	☐
2. I believe my child will develop spoken language similar to typically developing children their own age.	☐	☐	☐	☐	☐	☐
3. I believe my child will have greater educational opportunities and achievements.	☐	☐	☐	☐	☐	☐
4. I believe my child will need more of my daily attention than other (typically developing) children their own age.	☐	☐	☐	☐	☐	☐
5. I believe that as an adult my child will be able to find employment and support themselves.	☐	☐	☐	☐	☐	☐
6. I believe my child will lead a happy life.	☐	☐	☐	☐	☐	☐
7. I believe my child will feel safe and confident in the world.	☐	☐	☐	☐	☐	☐

**SECTION 2**<br>**Impact on Your Family**	Strongly Agree	Agree	Unsure	Disagree	Strongly Disagree	N/A
8. I always worry about whether the hearing device/s is/are working correctly.	☐	☐	☐	☐	☐	☐
9. Our financial situation is stressed by the ongoing costs to maintain the hearing device/s (e.g. spare parts, batteries, travel to appointments etc).	☐	☐	☐	☐	☐	☐
10. I devote more time to my child than other members of my family.	☐	☐	☐	☐	☐	☐
11. Our immediate family's activities are limited by our child's hearing needs.	☐	☐	☐	☐	☐	☐
12. The hearing device/s has/have improved communication between my immediate family members.	☐	☐	☐	☐	☐	☐
13. The future educational placement and achievement for my child is a concern for our immediate family.	☐	☐	☐	☐	☐	☐
14. We have had to change our working patterns since our child received their hearing device(s).	☐	☐	☐	☐	☐	☐
15. My child's hearing needs place additional stress on our immediate family.	☐	☐	☐	☐	☐	☐

**SECTION 3** **Quality of Life**	Strongly Agree	Agree	Unsure	Disagree	Strongly Disagree	N/A
16. My child is able to communicate their needs using spoken language.	☐	☐	☐	☐	☐	☐
17. My child is confident in social situations with typically developing children their own age.	☐	☐	☐	☐	☐	☐
18. My child initiates conversations with other children and adults.	☐	☐	☐	☐	☐	☐
19. My child's speech is clear and can be understood by people who have not met them before.	☐	☐	☐	☐	☐	☐
20. My child is often ill and asks to stay home from school.	☐	☐	☐	☐	☐	☐
21. My child overhears conversation that is not directed at them and asks questions about what was said.	☐	☐	☐	☐	☐	☐
22. My child keeps up with their peers for daily learning activities at pre-school/ kindergarten/school.	☐	☐	☐	☐	☐	☐
23. My child enjoys music.	☐	☐	☐	☐	☐	☐
24. My child tires more easily than other typically developing children their own age.	☐	☐	☐	☐	☐	☐
25. My child is often afraid of new situations.	☐	☐	☐	☐	☐	☐

Thank you for your co-operation!

Cochlear®
Hear now. And always

Cochlear, Hear now. And always, Nucleus, and the elliptical logo are trademarks of Cochlear Limited. © Cochlear Limited 2014
